# Facilitators and Barriers to Implementing Healthy School Canteen Intervention among Malaysian Adolescents: A Qualitative Study

**DOI:** 10.3390/nu13093078

**Published:** 2021-09-01

**Authors:** Nurul Ain Azizan, Angeliki Papadaki, Tin Tin Su, Muhammad Yazid Jalaludin, Shooka Mohammadi, Maznah Dahlui, Mohd Nahar Azmi Mohamed, Hazreen Abdul Majid

**Affiliations:** 1 Center of Population Health (CePH), Department of Social and Preventive Medicine, Faculty of Medicine, University of Malaya, Kuala Lumpur 50603, Malaysia; ain_azizan@usm.my (N.A.A.); tintin.su@monash.edu (T.T.S.); shooka.mohammadi@gmail.com (S.M.); maznahd@ummc.edu.my (M.D.); 2 Advanced Medical and Dental Institute, Universiti Sains Malaysia, Bertam 13200, Malaysia; 3 Centre for Exercise, Nutrition and Health Sciences, School for Policy Studies, University of Bristol, Bristol BS8 1TZ, UK; angeliki.papadaki@bristol.ac.uk; 4 South East Asia Community Observatory (SEACO), Jeffrey Cheah School of Medicine and Health Sciences, Monash University Malaysia, Bandar Sunway, Subang Jaya 47500, Malaysia; 5 Department of Paediatrics, Faculty of Medicine, University of Malaya, Kuala Lumpur 50603, Malaysia; yazidj@ummc.edu.my; 6 Department of Sports Medicine, Faculty of Medicine, University of Malaya, Kuala Lumpur 50603, Malaysia; nahar@ummc.edu.my; 7 Department of Nutrition, Faculty of Public Health, Universitas Airlangga, Surabaya 60115, Indonesia

**Keywords:** healthy eating, diet, school-based, adolescents, Malaysia

## Abstract

This study evaluated implementing a school-based intervention to promote healthier dietary habits in the school environment among Malaysian adolescents using qualitative methods. This qualitative study was conducted in four secondary schools in Perak and Selangor (two urban and two rural schools) that received the intervention (either training or training and food subsidy). A total of eight focus groups (68 students aged 15 years old) and 16 in-depth interviews were conducted with canteen operators, school convenience shop operators, school teachers and school principals in each school. Thematic analysis was used to analyse the qualitative data to identify suitable themes. We found several initiatives and changes by the schools’ stakeholders to change to a healthy school canteen programme. The stakeholders also noticed the students’ food preferences that influence healthy food intake in canteens and convenience shops. The food vendors and school administrators also found that subsidising healthy meals might encourage healthy eating. Among barriers to implementing healthy school initiatives were the student’s perception of healthy food and their eating habits, which also affect the food vendors’ profit if they want to implement a healthy canteen. The school-based intervention has the potential to promotes healthier eating among school adolescents. Continuous training and monitoring of canteen operators and convenience shops are needed, including building partnerships and educating the students on healthy eating to cultivate healthy eating habits.

## 1. Introduction

Globally, childhood obesity is becoming one of the major problems in public health, with one in every ten young people aged 5–17 being overweight or obese [1]. Middle and low-income countries are not exempted from this, with the prevalence of obesity among school children in Malaysia rising to 11.9% in 2015 from 6.1% in 2011 [2,3].

Obese adolescents consume more energy-dense diets that are high in sugar, oil, fat, and processed foods compared with adolescents who have a normal body mass index [4,5]. Particularly, male adolescents tend to have poor dietary intake high in sugar and salt with low consumption of fruits, vegetables and dairy products, and higher energy and macronutrient intake than female adolescents [6,7,8]. The Malaysian Health and Adolescents Longitudinal Research Team (MyHeART) study among Malaysian adolescents suggested that students who live in rural areas consumed more sugar, cholesterol and energy in their dietary intake than their peers in urban schools [4]. Several other studies also showed that Malaysian adolescents are likely to consume unhealthy foods and practice unhealthy eating behaviours [9,10]. Poor dietary intake, such as high intake of processed foods, soft drinks and confectionery, can significantly reduce performance in school, while intake of fruits, vegetables and milk can significantly improve curricular and co-curricular performance [11]. As more than 12% of adolescents in Malaysia are at high risk of developing cardiovascular disease (CVD) later in life, early interventions on healthy dietary intake and lifestyle modification are essential to reduce the progression of developing CVD in the future [12]. Intervention as early as in adolescent period is more effective than intervention during adult’s years, and this intervention in the long run will minimise the risks of developing non-communicable diseases in the future [13].

Schools are a strategic platform to cultivate healthy food intake behaviour and obesity prevention as students spend most of their time daily in school [14,15]. Providing healthy foods in school canteens can help improve children’s performance in school and educate them on the importance of healthy dietary intake in adulthood [11]. The school environment poses an opportunity to cultivate healthy eating habits among children and adolescents, as students spend at least six hours in school per day [16]. School meal programmes can be used as strategies to improve fruits and vegetable intake, while exposing children to various fruits and vegetables during school meals seems to help improve the intake of fruits and vegetables at home [17,18]. The recommendations on healthy eating, specifically for children and adolescents are included in the Malaysian Dietary Guidelines for children and adolescents [19]. Based on the guidelines, children and adolescents are encouraged to consume fruits, vegetables, milk and dairy products, as well as plenty of water daily [19]. It is important to introduce a targeted healthy dietary intervention programme for school children to improve their healthy food intake and preferences and reduce the prevalence of emerging non-communicable diseases later in life [2,4,20].

This study evaluated implementing a school-based intervention to promote healthier dietary habits in the school environment among Malaysian adolescents. The facilitators and challenges of implementing the intervention regarding its feasibility and acceptability are explored from several important school stakeholders, namely the school administrators, canteen operator, convenience shop operator and the students. This qualitative study is important to explore the stakeholder’s insights and opinions on the intervention to identify ways to improve the availability of healthy foods within the school environment and acceptance of the students on healthy foods.

## 2. Materials and Methods

### 2.1. Study Design and Participants

A qualitative study as part of the evaluation stage of a school-based intervention (two-arm, parallel-group, un-blinded, feasibility cluster randomised controlled study) was conducted within four schools in Selangor and Perak in Malaysia. This qualitative study was to evaluate the outcome from the MyHeARTBEaT (Malaysian Health and Adolescents Longitudinal Research Team Behavioral Epidemiology and Trial) project (IF017-2017) and was registered in the ISRCTN registry (ISRCTN 89649533). Prior to current study, a four-week feasibility intervention study was conducted in secondary schools located in Malaysia’s urban and rural areas involving two intervention arms. The intervention was developed based on data obtained from two related systematic reviews [6,21], reports of the MyHeART study [22] and a related qualitative study [23]. In addition, the framework for the development and evaluation of complex intervention by the UK Medical Research Council (MRC) was adapted for the current intervention [24,25]. This intervention study aimed to explore the feasibility of conducting an intervention in different types of schools (urban or rural) and its potential to show effectiveness for improving healthy eating practices in adolescents from different socioeconomic backgrounds. Four selected schools were divided into three groups, Intervention-1 and Intervention-2 groups. Each group has two schools (one from urban and one from a rural area). This intervention focused on providing healthier food options available in school, provided training to the food vendor operators in school, namely the canteen and convenience shop operators. The schools under Intervention-1 received training to prepare healthier food options for sale in the canteen and convenience shop. Meanwhile, the Intervention-2 group received similar training to Intervention-1 with the addition of a food subsidy. The food subsidy covered the cost of vegetables, fruits and low-energy dense *kuih* (traditional cake). The students involved in this intervention received food coupons to be used for the duration of one month. This coupon entitled them to get free vegetables, fruits and low-energy dense *kuih* that were subsidised for them at the canteen once a day during recess. Further details of the intervention are reported elsewhere [25]. Before the start of the intervention, the food vendor operators had received basic information on healthy canteens from the Ministry of Health (MOH) equally, which means that all of them had similar levels of information on healthy eating and a handbook about healthy canteens. This paper will discuss the intervention groups’ findings since we wanted to know the feasibility of conducting healthy canteen intervention and any potential effectiveness for the group that received training and group that received the training and food subsidy.

At the end of the intervention, 16 in-depth interviews were conducted with multiple stakeholders at the four schools, including the canteen operator, the school convenience shop operator, one teacher and one school principal at each school. Semi-structured questionnaire guides that were adapted and modified based on validated interview guides were used to conduct the interviews, which lasted 35 min per interview on average. The semi-structured interview guides were designed to seek the school administrators and food vendor operators’ views on the healthy canteen intervention programme and their views on the changes and barriers they discovered after the intervention programme. The questionnaire guides were developed based on a thorough systematic review and discussion among the teams and had been pre-tested [21,25]. A trained facilitator (MHA) experienced in conducting qualitative research facilitated the interviews which were conducted in Malay. The interviews explored stakeholders’ thoughts and views on the food environment changes and barriers encountered during the intervention.

At the same time, two focus groups were conducted in each of the four schools which had received intervention, involving students aged 15 years’ old. The focus groups were conducted separately for boys and girls in order to create a comfortable atmosphere where everyone feels free to give their ideas, express their thoughts without feeling restricted or intimidated by the presence of the opposite gender. The focus groups aimed to check on the availability of healthy food at the school and the school adolescent’s acceptance of the food environment. A semi-structured questionnaire guides were used to facilitate the discussion sessions among the students (online Supplemental Material). All participants (and the adolescents’ parent or legal guardian) were asked for written informed consent before the data collection. In total, eight focus groups from Intervention-1 and Intervention-2 arms were conducted by one trained facilitator and one note-taker conversing in Malay as the participants’ preferred. Focus groups lasted at least 40 min, with eight to ten students participating in each session.

### 2.2. Data Analysis

The audio-recordings were transcribed verbatim in Malay. Meanwhile, the translation to English was limited to selected quotes. This process was conducted to prevent misinterpretations of participants’ statements and fully consider the cultural context [26,27,28]. The focus group and interview transcripts were conducted by two trained researchers (NAA and KSJ) and verified by another researcher from the team. The inductive approach was conducted to form the initial coding and themes. Data analysis was conducted in inductive manner, a reflection of responses from the participants based on the questions and probes given that generated the codes and later the themes. The transcriptions were decoded using Microsoft Word and Microsoft Excel. Themes and quotes were translated into English by NAA and KSJ, who are native Malay speakers and back-translated to Malay by an independent bilingual researcher (MHA). Representative quotes are presented in English and Malay.

Thematic analysis was used to analyse the transcripts, using the framework method approach to generate the themes [29,30,31]. This framework approach was used as it can help to identify the similarities and differences in data collected and draw relationships between various parts of the data when identifying suitable themes by analysing inductively [30,32]. There are several steps in using thematic analysis to evaluate the data [31,33,34]. The first step was a transcription of the audio-recordings by the researchers and familiarisation with the data by reading the data repeatedly. After that, two trained researchers separately coded the transcripts to make it easy to classify and group the data. Coding inconsistencies were discussed between the researchers until a set of codes that made the initial framework was agreed upon. The inter-rater agreement was 90% by dividing the number of agreements by the sum of agreement and disagreement between the two coders’ set of codes. Then the initial framework was applied to all transcripts systematically using Microsoft Word and Microsoft Excel and further checked using ATLAS.ti. for Windows [35]. This step simplified the comparison of similarities and differences within and between the focus groups and in-depth interviews according to each theme. The data was then summarised by category from each transcript. Themes and sub-themes were supported by quotations from the participants (based on different stakeholders and groups of intervention) to reflect the diversity of their response.

## 3. Results

Eight focus group discussions and 16 in-depth interviews were conducted on the intervention groups, with a total of 68 adolescents (34 boys; 34 girls) and 16 multiple stakeholders, respectively (Table 1). The thematic analysis revealed several themes around the acceptability and barriers after implementing the feasibility study from the stakeholders and adolescents’ point of view. The results reflect the outcomes from the schools involved in the intervention. The summary of findings of acceptance, changes, and barriers encountered during the intervention are shown in Table 2, Table 3 and Table 4 while Table 5 shows the participants’ themes and responses.

### 3.1. Initiatives/Changes to Healthy School Canteen Programme

The feasibility study concluded with mixed opinions on the changes that were implemented in the canteen and convenience shops. Most of the students noticed the increased food choices in both the canteen and convenience shop. However, they perceived that the availability of healthy foods was still low. The canteen and convenience shop operators claimed to have stopped selling energy-dense foods and noticed a decrease in their sale profits. By reorganising the sugar-sweetened beverages (SSBs) in the drink section in the convenience shops, the sale of non-sweetened beverages had increased, with most students preferring to buy mineral water and juice. The convenience shop in the intervention schools also took the initiative to sell fruits to students. Most students in Intervention-2 used the provided food coupons, while some reported that they continued eating vegetables outside the school even after the program has ended. One school started the programme of providing healthy and budget meals for low-income students during recess.

### 3.2. Subsidy/Coupons for School Meals

The principals, canteen operators and convenience shop operators agreed that subsidising healthy meals encouraged healthy eating in schools. The canteen operators and school administrators thought that prepaying for meals might incentivise them to prepare healthy food for students as this strategy would reduce food wastage and lead to profit. Students in Intervention-2 reported that they used the coupon provided to buy vegetables, fruits and low-energy dense *kuih* at the canteen. As mentioned above, some students noticed that they had continued eating vegetables and fruits even after the programme ended. However, two school administrators complained that the students sometimes forgot to bring the coupon to claim their food subsidy.

### 3.3. Food preference/Acceptance among Students

The canteen operators in Intervention-2 noticed that the students have their own preferences when it comes to vegetables and fruits. Some vegetables and fruits were popular among students such as baby *kailan* (kale), guava and watermelon. Some of the female students in Intervention-2 reported not taking the *kuih* provided as they perceived that the healthy *kuih* provided is only suitable for adult taste. Most of the intervention group students stated that they would likely buy healthier options if these were available in the canteen, as they think healthy food is good for their body and health. Students from the control group said that they would likely buy healthy foods from the canteen, provided they are cheap and tasty. However, canteen operators and school administrators in both Intervention 1 and 2 both thought that students preferred unhealthy options compared to healthier ones and will not spend money on foods they do not like.

### 3.4. Barriers to Implementing the Intervention in the School Canteens

Most of the students thought that the taste and high price of healthy foods were barriers to healthy eating in school. They thought that healthy foods should taste sour, with no added salt (tasteless), which they did not like. Healthy foods were perceived as expensive, and not all students will buy. Meanwhile, the canteen operators thought that students already have their eating habits and preferences that would not be easy to change. The canteen operators in both Intervention-1 and Intervention-2 were familiar with the healthy canteen guidelines as it was similar to the guidelines from the Ministry. However, they found it hard to comply due to cost and profit, especially perishable items such as vegetables and fruits. Besides, they noticed students like to buy energy-dense foods, which are more profitable for their sales. They suggested that healthy eating intervention should start earlier at home and primary school level to make it easier to change and adapt to healthy foods. The canteen operators also identified that lack of manpower in preparing healthy meals might become an obstacle in implementing healthy canteen.

## 4. Discussion

This intervention study aims to evaluate the implementation of healthy canteens in schools. The findings managed to capture the opinions and insights from school important stakeholders, namely the school administrators, food vendor operators and students. The information on the challenges, barriers and suggestions on healthy canteen implementation were also collected. Canteens and convenience shops are the primary sources of food for children in school. The foods sold in the school contribute up to 50% of the students’ daily calories intake. Abundant low-nutrition, energy-dense foods sold in the school can easily be the reason for malnutrition among school children [5,14]. Despite the guide on healthy canteens by the Ministry of Education, one-third of the food sold are the processed foods, still being sold in the school canteen, with limited availability of fruits, vegetables and milk [36]. Hence, it is crucial to highlight the importance and responsibility of food vendors in school to help create a healthy food environment and provide balanced and nutritious food for the students. Continuous training to food vendors operators in school could reduce RED (energy-dense foods) items sold in the canteen and convenience shops in schools [37]. Providing healthier options in the canteen and convenience shops and eliminating unhealthy options can encourage students to start practicing on healthy dietary intake [38].

From this study, the principal and teachers noticed an increase in the canteen’s healthier options and changes in student’s food intake during intervention. Providing subsidies for healthy meals such as fruits, vegetables, and low-energy dense delicacy at the canteen promotes and educates healthy eating at school and might reduce possibilities of any stigma or feeling of embarrassment in changing their dietary intake towards healthier options [23,39]. Providing food subsidies can help reduce the cost of the canteen operators in preparing healthy foods for students. In fact, some stakeholders suggested providing food subsidies as one of the strategies to improve healthy eating among school adolescents [23]. Providing prepaid meals was also one of the operators’ suggestions in this study as it can reduce food wastage, with similar healthy food pre-order and packed for all students. Having similar meals together can give positive peer support in practising healthy dietary intake daily [16,39]. Besides providing option for healthy prepaid meals that parents can choose for their children in school, it shows that involving parents in cultivating a healthy food environment in the school is very important [39]. Including parents in nutrition education in school, whether direct or indirectly might encourage their children to practice healthy eating behaviour, even outside of school [40].

This study shows that the students in both intervention groups noticed some changes in their dietary habits after the intervention. There is better acceptance of mineral water, yoghurt, and juices at the convenience shop when they rearrange the drinks according to mineral water first followed by milk, yoghurt, juices and SSBs. By providing healthy options in school convenience shops can positively influence the healthy food perceptions of the students [41]. Adolescents preferred healthy dietary intake promotion strategy rather than discouraging unhealthy dietary intake strategy. Girls, young and overweight adolescents were better in accepting health intervention than their counterparts [38]. For current study, most of the intervention groups’ students noticed changes in their dietary intake after the end of the intervention phase, especially group received subsidy on foods. Adolescent adaptation to healthier food and lifestyle is easier if it was done together with friends [16]. This is because they tend to consume the same foods together and do not feel disadvantage of consuming different foods, which makes it a perfect setting of introducing and cultivating intake of healthy foods in school. In addition, the students in Intervention-2 who received a subsidy on fruits, vegetables and less energy-dense *kuih* noticed changes in their food habits after the intervention and more concern about the expensive price as a barrier for healthy eating at school, compared with intervention group without food subsidy who were more concerned on the taste of healthy foods as barrier to setting up healthy canteen. From this finding it shows that the students can accept the taste of healthy food if it is prepared properly. Adolescents can accept or start to like healthy food when their peers are consuming the same food [16,39].

Lack of food variety and hygiene usually highlighted by students as barrier for healthy school canteen implementation [42]. In this study, most of the students in both intervention groups highlighted the availability of foods in a convenience shop at school that was mostly a mixture of healthy and unhealthy foods. This shows that most of the students can differentiate between the healthy and non-healthy snacks sold in convenience shops. However, when asked about the type of healthy food they consumed at the school, some cited ‘*roti canai*’, ‘*roti coklat*’, nugget and sausages as healthy. This is similar to the finding of the previous study that the students perceived oily foods as “healthy” [23]. This shows that it is important not only to educate the school food providers on healthy eating but also the students.

Meanwhile, food preferences among the students can also be a barrier in the acceptance of the implementation of healthy food in school. Familiarity with the taste is among the reasons for them to accept and eat the food [43]. This shows that it is important to start cultivating healthy eating habits early to be easier to implement healthy food environment in school. Students tend to perceive healthy foods are not tasty, probably due to the different cooking methods or modification that might make it unappealing or tasteless to them [43]. School administrators also perceived student’s usual food intake and taste preferences as barriers toward healthy canteen implementation, which usually start from home [44]. Decreasing in sales was one of the main problems for the food vendors when energy-dense foods and low-nutrition foods were minimised from the menu during intervention period in this study. This was also one of the reasons for the school food vendors’ reluctance to implement the healthy canteen initiative [14]. However, removing or reducing the RED foods consistently in the school may increase students’ acceptance of healthy and balanced food options to augment the loss experienced by the food vendors [45]. In this study, the canteen operators also perceived that limited manpower was an important barrier for the future implementation of such interventions. This might be due to their perception that preparing healthy foods, especially fresh food sources, will consume more time and energy [46]. This might suggest further education and monitored enforcement to the food providers to ensure smooth implementation of school healthy canteen.

### Strengths and Limitations of the Study

This is the first qualitative study that focuses on several stakeholders at the secondary school set up, including students, food providers, and school administrators on their acceptance and barriers to healthy eating intervention in school. This is one of the strengths of this study. A wide range of stakeholders was involved with large sample sizes recruited from both urban and rural schools in Malaysia across several districts, thus providing immense insights and views regarding the school-based intervention on healthy eating. Their diverse inputs regarding the topic might help prepare a more holistic approach in implementing a healthier food intervention in school settings. However, the students may be influenced by their friends’ answers during the FGD session, especially when they need to provide an opinion that probably does not reflect their thoughts and views.

## 5. Conclusions

The school-based intervention has the potential to be a platform to promote healthier eating among school adolescents. Students improved their acceptance of fruits and vegetables when consumed daily. Continuous training and monitoring of canteen operators and convenience shops are needed, not only by the school administration but also from the responsible ministry. Partnerships with them from the start is crucial to foster healthy eating habits and environment. In addition to providing education and giving training to canteen and convenience shop operators, educating the students on healthy eating is critical to cultivate the habits of eating healthily, not only at school but also in their daily life. Further intervention evaluation is required to see the impact of the healthy changes made.

## Figures and Tables

**Table 1:** Participation in Focus Group Discussions and Interviews.

Schools	Focus Groups (*n*:68)	In-Depth Interviews (*n*:16)
Intervention 1-urban school	Boys (8)	Principal (1)
	Girls (8)	Teacher (1)
		Canteen operator (1)
		Convenient shop operator (1)
Intervention 1-rural school	Boys (8)	Assistant Principal (1)
	Girls (8)	Teacher (1)
		Canteen operator (1)
		Convenient shop operator (1)
Intervention 2- urban school	Boys (9)	Assistant Principal (1)
	Girls (9)	Teacher (1)
		Canteen operator (1)
		Convenient shop operator (1)
Intervention 2- rural school	Boys (9)	Assistant Principal (1)
	Girls (9)	Teacher (1)
		Canteen operator (1)
		Convenient shop operator (1)
Total	8 focus groups 34 boys (50%) 34 girls (50%)	16 in-depth interviews Teacher (Male 0%; Female 83%) Principal (Male 50%; Female 50%) Canteen operator (Male 75%; Female 25%) Convenience shop operators (Male 25%; Female 75%)

**Table 2:** Acceptability, Challenges and Barriers Towards Healthy Eating at School (Intervention Arms 1 and 2 *).

	School Managements (Principal or Assistant Principal)	Canteen Operators	Convenient Shop Operators	Teachers
Acceptability of this programme	-noticed some changes in canteen and convenience shop -noticed more vegetables sold in canteen -noticed healthier food options in convenience shop. -noticed that students start to change eating habit	-students have their own food preferences -students like selected vegetables and fruits -school give full support	-students have their own food preferences -start to sell fruits in convenience shop -buying behaviour changes due to availability of food in convenience shop -acceptance in changes of non-sweetened beverages -good programme/learn a lot -good to have detailed guideline for healthy school -students suggest more healthy foods to sell	-notice little changes in canteen and convenience shop (e.g., less unhealthy food and fruits sold in canteen and convenience shop) -good programme/full support -convenience shop has both healthy and non-healthy option
Challenges during intervention	-students can still buy unhealthy food outside school -students worried of other perception if drink plain water -students dislike healthy beverages	-students did not like vegetables -lack of students’ understanding of healthy food -students’ usual eating habit (lack of vegetables and fruits) -not see many changes due to short duration -cost of food preparation -sales drop when stop selling energy-dense foods -students can still get energy-dense, fast food outside of school -canteen try to stop selling junk foods, but shops still sell it.	-limited variety of food to sell (cannot sell fresh foods) -clashes with canteen in terms of food/beverages to sell -students still prefer sweets beverages/unhealthy foods -students can still buy junk food outside of school -healthy choice of food is expensive -drop in sales	-lack of vegetables in canteen (students dislike vegetables) -students still like energy-dense foods -canteen and convenience shop still sell unhealthy food -lack of students’ understanding on healthy eating
Barriers for future implementation	-students hard to accept vegetables -students can still buy unhealthy food outside school -students only buy what they like to eat; mostly unhealthy foods -lack of health awareness of students -unhealthy dietary intake since young	-high cost of preparing healthy food -facing loss if fully sell healthy food -students’ preferences on healthy food intake, need to educate them -education on healthy food	-limitation of type of food sold in convenience shop	-lack of understanding on healthy foods (from students and canteen operators) -limited choice of healthy foods -lack of health awareness of students
Suggestions to improve healthy eating	-encourage continuation of this programme -implementation should involve everyone, not only school canteen -consider students’ food acceptance (preference? to change eating habit	-prepay meals for students -healthy food intervention should start early (primary school/home)	-focus on obese students -focus on foods that are good for students’ energy and wellbeing -subsidise healthy foods -teacher’s responsibility to develop healthy environment -continue programme/healthy campaign	-continue collaboration school and canteen -teacher’s responsibility to develop healthy environment -healthy food campaign

* Intervention-1: Training only. Intervention 2: Training and meals subsidy.

**Table 3:** Changes, Challenges and Barriers to Healthy Eating at School after the End of The Intervention (Intervention Arms 1- training only).

	Boys (Intervention 1-Rural)	Girls (Intervention 1-Rural)	Boys (Intervention 1-Urban)	Girls (Intervention 1-Urban)
Changes in canteen	***Healthier changes*** -less snacks and sweet beverages ***Least/unhealthy changes*** -none noticed	***Healthier changes*** -increase in food variety ***Least/unhealthy changes*** -more energy-dense foods -increase in food variety including deep fried food, bun, fries, ice-cream	***Healthier changes*** -increase in food variety -food sold with less oil, salt and sugar -more hygienic than before -price of food cheaper	***Healthier changes*** -increase in food variety -food choice with less oil -food is cheaper than before -healthier with added vegetable
Changes in convenience shop	***Healthier changes*** -selling can drinks, bottled fruit juices, yogurt drink ***Least/unhealthy changes*** - more energy-dense foods (snacks)	***Healthier changes*** -less SSBs ***Least/unhealthy changes*** -more energy-dense foods (snacks) -variety of fruit juices, yogurt drink, ice-cream, snacks, bread	***Healthier changes*** -more fruits and healthier options ***Least/unhealthy changes*** -more snacks such as milo nugget and biscuit, sweet, sausage bread	***Healthier changes*** -more fruits and healthier choices -variety of fruits, snacks and bread -food is cheaper than before
Changes in food habits	-none noticed	-none noticed	-none noticed	-able to accept vegetables taste if cooked nicely
Challenges faced during intervention	-none noticed	-none noticed	-less healthy food option at the canteen -healthy food is unattractive	-less healthy food option at the canteen -unhealthy food taste better
Barriers for future implementation	-healthy food maybe not tasty -healthy food might be expensive -healthy food means decrease in portion size	-healthy food maybe not tasty	-hard to change eating habits of the student -healthy food means decrease in portion size	-healthy food maybe not tasty (sour) -hard to change eating habits of the students

**Table 4:** Changes, Challenges and Barriers to Healthy Eating at School after the End of The Intervention (Intervention Arms 2- training with food subsidy).

	Boys (Intervention 2-Rural)	Girls (Intervention 2-Rural)	Boys (Intervention 2-Urban)	Girls (Intervention 2-Urban)
Changes in canteen	***Healthier changes*** -use the coupon and take the foods provided. -can save money and eat healthy food using coupon ***Least/unhealthy changes*** -no obvious changes in canteen -should add more variety of food, fruits, vegetable	***Healthier changes*** - use the coupon and take the foods provided. ***Least/unhealthy changes*** -no obvious changes in canteen -should reduce oil in cooking -only take *kuih* and fruits that suitable to their taste -vegetable dishes too salty	***Healthier changes*** -more food choice -good programme -use coupon provided ***Least/unhealthy changes*** -lack of hygiene -not informed on free drink provided -most did not take vegetables	***Healthier changes*** -increased in food variety -increase of healthy food choices - programme helps to practice habit of eating healthy ***Least/unhealthy changes*** -food oilier than previous canteen operator -lack of hygiene - not informed on free drink -most take fruits and *kuih* than vegetable
Changes in convenience shop	***Healthier changes*** -more food variety -healthier option than canteen -more mineral water/yogurt than SSBs ***Least/unhealthy changes*** -addition of ice-cream -has both healthy and unhealthy food on sale	***Healthier changes*** -no changes ***Least/unhealthy changes*** -addition of ice-cream for sale	***Healthier changes*** -no changes ***Least/ unhealthy changes*** -no changes	***Healthier changes*** -no changes ***Least/ unhealthy changes*** -no changes
Changes in food habits	-mostly think they eat healthily than before -increased in likeness to vegetables, fruits and *kuih*	-changes in healthier food habits outside school -increased in fruits and vegetables intake -start to like to eat *kuih*, fruits and vegetable	-take healthier food during the programme	-take healthier food during the programme -continue to eat fruits after programme ended
Challenges faced during intervention	-food too oily at canteen -beverages too sweet	-healthy food is expensive -dishes sold too oily, too salty or too spicy -lack of fruits and vegetables in menu -lack of variety in menu	-lack of food hygiene in canteen -not satisfied with taste of food provided	-food too oily at canteen -more choice of unhealthy food at canteen -not satisfied with food taste in canteen
Barriers for future implementation	-healthy food might be expensive	-none	-healthy food might be expensive -concern on hygiene and cleanliness of food preparation	-healthy food might be expensive -healthy food means decrease in portion size -concern on cleanliness and hygiene of food preparation in canteen

**Table 5:** Acceptance and Challenges to The Implementation of School-Based Intervention to Improve Healthy Eating Practices Among School Adolescents.

Theme/Subtheme	Example of Responses
Initiatives/changes to healthy school canteen programme	“for me, I have change..mmm I have start to like to eat vegetables, have start to like vegetables and fruits..” (F8, Intervention 2, rural) ” aa..canteen did sell sweetened beverages but not no more” (B4, Intervention 1, rural). “aaa from the programme, the foods served quite nutritious such as vegetable, fruits and *kuih*. I took the food served form the programme everyday..and now I have come to like the vegetables and *kuih-kuih* “ (B3, Intervention 2, rural) “yes…we can see increased in menu..aah..to healthier menu and not fast food to us, we cook..use the fresh ingredients and sell to students “ (Co-op operator, Intervention 2, urban)
Barriers to healthy school canteen implementation	“I think in terms of taste..probably sour..the price might be expensive and small in quantity (B3, Intervention 1, rural) “aa.. in terms of student acceptance? Because students are used to greasy food like that, because..then they are afraid that students will not be able to accept this..(F3, Intervention 1, rural) “have to hire more workers” (Canteen operator, Intervention 2, urban) “if we do not sell at canteen (energy-dense foods), the others will sell it…the students still can buy it..outsides school..besides if following the guidelines there are certain distance (to sell)..”(canteen operator, Intervention 1, rural)
Foods preferences/acceptance among students	“I will buy (if canteen served healthy food), because less oily, less fat and quite good for health” (B2, Intervention 2, urban) “okay, mmm in my opinion, I will buy if the canteen has changed to a healthy canteen because it may reduce the fat in my body, in terms of oil, after that..haa can keep my body healthy and I will take food, buy food at the canteen if you have changed it to healthy” (F4, Intervention 1, rural) “it is a bit fussy among the students, students they want. they want sweet things, delicious food, because as a seller, so if I can sell then I will get profit, more profit to me, but because we have guidelines so I have to follow” (Canteen operator, Intervention 1, rural) “ah for example milk, many students will buy but more to flavoured milk. Many students, but certain students they will buy milk everyday” (Assistant Principal, Intervention 1, urban)
Subsidy/coupon for healthy foods	“for me (giving food subsidy) is really helpful, because indirectly, maybe the student does not like to eat vegetables, he does not like to eat vegetables but with the subsidy, he will feel the coupon will be wasted (if he did not use). eventually he will try to eat and interested to eat vegetables..” (Principal, Intervention 2, urban)” I took the provided foods and I feel that I can save money, and the food that I take is healthier than others “(B1, Intervention 2, rural) “in my opinion it is good because students can eat healthy food, and can eat everyday those things that they don’t usually eat such as fruits, in my opinion.” (B1, Intervention 2, urban) “it is good because actually students like free things” (Teacher, Intervention 2, urban) “well no (profit will not affected), in fact it is better, because even if students eat or not, the meal already paid” (Canteen operator, Intervention 2, urban)

## Supplementary Materials

The following are available online at https://www.mdpi.com/article/10.3390/nu13093078/s1, (a), Questionnaire guide (b), Examples of responses in English and Malay language.
