# Supplementary material for: Facilitators and Barriers to Implementing Healthy School Canteen Intervention among Malaysian Adolescents: A Qualitative Study
Source: Nutrients. 2021 Sep 1;13(9):3078. doi: 10.3390/nu13093078 (PMC8471853; doi:10.3390/nu13093078)
Supplement: Supplementary file 1 [file nutrients-13-03078-s001.zip › nutrients-1365626-supplementary.pdf]

Supplementary material

a) Example of Semi-structured Questionnaire Guide

1. Have you noticed any changes in the school canteen?

A) what sort of changes?

B) what do you think of these changes?

2. Do you aware of the implementation of the Healthy School Canteen programme? If yes, what has been your experience from the implementation?

3. How would you describe the foods that are available in the canteen?

4. What do you think on the new environment of the canteen vs. The one, one month ago?

5. Do you think your purchases have changed over the last month? In what way? Examples.

6. Have you noticed the water dispenser in the school? Have you been using it?

7. What do you think of the coupons you have been provided about fruit and *kuih*? Did they help you purchase more fruit and *kuih*? Why? / why not?

8. Are there more items categorized as healthy food items on the menu no, prior to implementation? If yes, please give examples.

9. Are you interested of buying healthy options? Why/ why not?

10. What foods and beverages do you eat just about everyday that you think **healthy**? From the answer number 3, would you like to buy the food and drinks on a daily basis?

11. What foods and beverages do you eat just about everyday that you think are NOT HEALTHY? What is the unhealthy food you consumed daily?

12. Did you face any challenges for buying healthy foods? If yes, what are the challenges?

13. What suggestions would you make to your school to promote healthy eating? What would you suggest to make the school canteen a better place to buy healthy food? Do you like this program?

b) The examples of responses related to Acceptance and Challenges to The Implementation of School-Based Intervention to Improve Healthy Eating Practices Among School Adolescents based on the themes (English and Malay version)

Initiatives / changes to healthy school canteen program

- "for me, I have change..mmm I have start to like to eat vegetables, have start to like vegetables and fruits.." (F8, Intervention 2, rural) / *"mmm pada saya , saya ada perubahan. Mmm..perubahan saya, saya dah sekarang ni dah banyak makan sayur, mula suka sayur, lepas tu mula suka buah"* (F8, Intervensi 2, luar bandar)

- " aa..canteen did sell sweetened beverages but now no more" (B4, Intervention 1, rural) / *aa dulu kat kantin ada jual air kotak tapi sekarang tak jual dah.*

- " aaa from the program, the foods served quite nutritious such as vegetable, fruits and kuih. I took the food served form the program everyday..and now I have come to like the vegetables and kuih-kuih " (B3, Intervention 2, rural)/ *aaa yang program yang dibuat oleh sekolah itu makanan nya agak berkhasiat seperti sayur, buah-buahan dan kuih. Yang dapat kami makan dan sayur tersebut telah kami ambil.saya ambil hari – hari sepanjang program tersebut dan..saya kini telah suka, agak suka terhadap sayur-sayuran dan kuih-kuih*

- " yes…we can see increased in menu..aah..to healthier menu and not fast food to us, we cook..use the fresh ingredients and sell to students " (Co-op operator, Intervention 2, urban)/ *ah, kepada menu-menu yang agak sihat dan kepada yang kita bukan makanan segera, kita masak.. guna yang fresh, dan menghasilkan menu dan jual kepada pelajar*

Barriers to healthy school canteen implementation

- "I think in terms of taste..probably sour..the price might be expensive and small in quantity (B3, Intervention 1, rural) / *I think dari segi rasa. Rasa mungkin masam..harga nya mungkin lebih mahal.and then kuantiti mungkin kurang*

- "aa.. in terms of student acceptance? Because students are used to greasy food like that, because ..then they are afraid that students will not be able to accept this..(F3, Intervention 1, rural) / *aa ni kot daripada segi penerimaan pelajar? Sebab pelajar kan dah biasa dengan makanan yang berminyak macam tu kan, sebab ..lepas tu takut pelajar tak dapat terima apa yang ni lah, kempen ni*

- “have to hire more workers” (Canteen operator, Intervention 2, urban) / *kena lebih pekerja lah*

- “ if we do not sell at canteen (energy-dense foods), the others will sell it…the students still can buy it..outsides school..besides if following the guidelines there are certain distance (to sell)..”(canteen operator, Intervention 1, rural) / *kalau kami tak buat kat kantin, ada pihak lain yang buat..mereka masih boleh dapatkan..satu lagi..di luar-luar lah..bahagian luar .lagipun kalau garis panduan kalau ikut ada berapa meter, berapa meter kan..di sini ada..kat sana situ*

Foods preferences/acceptance among students

- “ I will buy (if canteen served healthy food), because less oily, less fat and quite good for health” (B2, Intervention 2, urban) / *B2: maybe saya akan beli sebab makanan dia kurang berminyak, kurang berlemak, agak sihat untuk kesihatan*

- “okay, mmm in my opinion, I will buy if the canteen has changed to a healthy canteen because it may reduce the fat in my body, in terms of oil, after that..haa can keep my body healthy and I will take food , buy food at the canteen if you have changed it to healthy” (F4, Intervention 1, rural) / *okey, mmm pada pendapat saya , saya akan beli kalau kantin tu dah ubah kantin yang sihat sebab mungkin dapat mengurangkan lemak-lemak dalam badan saya, dari segi minyak kan, lepas tu..haa dapat menyihatkan badan saya dan saya akan ambil makanan , beli makanan kat kantin kalau dah ubahkan dengan sihat*

- “it is a bit fussy among the students, students they want.. they want sweet things, delicious food, because as a seller, so if I can sell then I will get profit, more profit to me, but because we have guidelines so I have to follow” (Canteen operator, Intervention 1, rural) / *benda tu agak cerewetlah daripada segi pelajar tu, pelajar diorang nak, nak yang manis, nak yang sedap..sebab saya peniaga ni, jadi kalau dapat jual tu untunglah, untung kat saya lah tapi sebab ada garis panduan semua jadi saya terpaksa ikut lah*
-
- “ah for example milk , many students will buy but more to flavoured milk..many students, but certain students they will buy milk everyday” (Assistant Principal, Intervention 1, urban) / *ah macam susu tu memang bila, ah, budak banyak beli lah, tapi lebih kepada susu ber-flavor...ada lah, memang banyak budak, ada certain budak memang hari-hari dia nak susu*

Subsidy/coupon for healthy food

- "for me (giving food subsidy) is really helpful, because indirectly, maybe the student does not like to eat vegetables, he does not like to eat vegetables but with the subsidy, he will feel the coupon will be wasted(if he did not use)..eventually he will try to eat and interested to eat vegetables.." (Principal, Intervention 2, urban) / *ah, memang lah membantu, sebab itu secara tak langsung, mungkin murid tu dia tak suka makan sayur kan, dia tak suka makan sayur, tapi dengan subsidi tu, nanti dia membazir dia punya kupon tu. dia akan cuba makan kan kali pertama tu, tak minat sangat, lama-kelamaan dia akan minat lah makan sayur tu...*

- " I took the provided foods and I feel that I can save money, and the food that I take is healthier than others "(B1, Intervention 2, rural) / *saya mengambil makanan yang disediakan dan saya rasa perubahan yang saya dapat rasakan ialah saya dapat menjimatkan duit dan makanan yang saya mengambil, yang saya ambil lebih sihat berbanding aa yang selain*

- "in my opinion it is good because students can eat healthy food, and can eat everyday those things that they don't usually eat such as fruits, in my opinion.." (B1, Intervention 2, urban) / *erm, pada pendapat saya bagus lah sebab murid-murid dapat mengamalkan makanan sihat tu, lepas tu ah dia dapat makan setiap hari lah yang mana tak pernah makan buah tu makan, pendapat saya…*

- "it is good because actually students like free things" (Teacher, Intervention 2, urban)/ *ah bagus sebenarnya sebab budak sebenarnya kalau ikutkan budak suka beli benda yang free*

- "well no (profit will not affected) , in fact it is better, because even if students eat or not, the meal already paid"(Canteen operator, Intervention 2, urban) / *tak lah, lagi bagus. Dah tentu, ah, dia makan tak makan, dia dah bayar*
